# DR-IIXRN : Detection Algorithm of Diabetic Retinopathy Based on Deep Ensemble Learning and Attention Mechanism

**DOI:** 10.3389/fninf.2021.778552

**Published:** 2021-12-24

**Authors:** Zhuang Ai, Xuan Huang, Yuan Fan, Jing Feng, Fanxin Zeng, Yaping Lu

**Affiliations:** ^1^Department of Research and Development, Sinopharm Genomics Technology Co., Ltd., Jiangsu, China; ^2^Department of Ophthalmology, Beijing Chao-Yang Hospital, Capital Medical University, Beijing, China; ^3^Medical Research Center, Beijing Chao-Yang Hospital, Capital Medical University, Beijing, China; ^4^Department of Clinical Research Center, Dazhou Central Hospital, Sichuan, China

**Keywords:** diabetic retinopathy, image processing, ensemble learning, deep learning, attention mechanism

## Abstract

Diabetic retinopathy (DR) is one of the common chronic complications of diabetes and the most common blinding eye disease. If not treated in time, it might lead to visual impairment and even blindness in severe cases. Therefore, this article proposes an algorithm for detecting diabetic retinopathy based on deep ensemble learning and attention mechanism. First, image samples were preprocessed and enhanced to obtain high quality image data. Second, in order to improve the adaptability and accuracy of the detection algorithm, we constructed a holistic detection model DR-IIXRN, which consists of Inception V3, InceptionResNet V2, Xception, ResNeXt101, and NASNetLarge. For each base classifier, we modified the network model using transfer learning, fine-tuning, and attention mechanisms to improve its ability to detect DR. Finally, a weighted voting algorithm was used to determine which category (normal, mild, moderate, severe, or proliferative DR) the images belonged to. We also tuned the trained network model on the hospital data, and the real test samples in the hospital also confirmed the advantages of the algorithm in the detection of the diabetic retina. Experiments show that compared with the traditional single network model detection algorithm, the auc, accuracy, and recall rate of the proposed method are improved to 95, 92, and 92%, respectively, which proves the adaptability and correctness of the proposed method.

## 1. Introduction

The number of people suffering from diabetes in China is now the first in the world. Studies have found that (Li Y. et al., 2020), from 2007 to 2017, the prevalence of diabetes in China has gradually increased over 10 years and has reached 12.8% in 2017, while the estimated prevalence of prediabetes was up to 35.2%, which indicates that diabetes is an important health problem in China. Diabetic retinopathy (DR) is one of the common chronic complications of diabetes and is also the most common blinding eye disease. In the early stages of the disease, patients may not feel anything unusual, but as the disease progresses, diabetic retinopathy can lead to vision impairment and possibly blindness. DR can be broadly classified into five stages: normal, mild, moderate, severe, and proliferative DR. Typically, an experienced physician will review fundus images to determine the current stage of DR the patient is in. However, doctors in different regions differ greatly, so it is difficult to guarantee the diagnosis, correctness. In remote areas, there may be no relevant doctors, making it impossible to detect such cases. Therefore, we need to use medical image recognition machines to help diagnose this disease. In the real world, the data set of DR is often extremely unbalanced. How to train the medical image recognition machine from the unbalanced data set and find the real patient has always been a research hotspot (Lian et al., 2018; Maistry et al., 2020).

Transfer learning is to transfer the trained model parameters to the new model to help the new model training (Torrey and Shavlik, 2010; Guo et al., 2021). Transfer learning can significantly improve the performance of the model (Kermany et al., 2018). Lu S. et al. (2021) proposed a new end-to-end novel Coronavirus classification system, neighboring aware graph neural network (NAGNN). In order to obtain good image-level representation, it uses transfer learning in the backbone neural network to acquire features, thus accelerating the training efficiency of the network. Lu S. Y. et al. (2021) proposed a new computer-aided diagnostic method for the Cerebral Microbleed test. First, a 15-layer FeatureNet is trained to extract features from input samples. Second, the structures after the first fully connected layer in FeatureNet are replaced by three random neural networks for classification: Schmidt neural network, random vector functional-link net, and extreme learning machine. In the training process of these three classifiers, the weight and deviation of FeatureNet's early layers are frozen. Finally, the outputs of the three classifiers are integrated through a majority voting mechanism to obtain better classification performance. Narin et al. (2021) proposed to use five types of pre-trained convolutional neural network (CNN) models (ResNet50, ResNet101, ResNet152, InceptionV3, and Inception-ResNetV2) to detect patients infected with coronavirus pneumonia based on chest X-ray. The pre-trained ResNet50 model obtained the highest classification performance. Ardakani et al. (2020) compared 10 famous CNN: AlexNet, VGG-16, VGG-19, SqueezeNet, GoogleNet, Mobilenet-V2, ResNET-18, RESnet-50, Resnet-101, and Xception's classification performance in differentiating infections in COVID-19 and non-COVID-19 groups. Of all the networks, Resnet-101 and Xception performed the best.

Compared with a single algorithm, ensemble learning methods often combine the output of multiple classifiers to achieve better performance (Chen et al., 2019; Minetto et al., 2019; Zheng et al., 2019), and ensemble learning based on CNN has been extensively studied in the medical field (Lin et al., 2021). Fu et al. (2018) proposed a novel disc-aware ensemble network for automatic screening of glaucoma, which integrates the deep hierarchical context of the global fundus image and the local optic disc region. Considered as a global image stream, segmentation guidance network, local disc region stream and disc polar transformation stream as four branches. Finally, the output probability of different branches is fused as the final screening result. Xiao et al. (2019) preferentially selects six models, DenseNet121, ResNet101, SENet154, VGG16, DeepTEN, and InceptionV4, as base classifiers. Then, he takes the average of the predictions from all the models and uses them to make the final prediction. Das et al. (2021) proposed a chest X-ray image detection of COVID-19 patients based on deep CNN, using multiple state-of-the-art CNN models that can make independent predictions after individual training. The models are then combined to predict class values using a new method of weighted average integration techniques. The commonly used ensemble learning fusion method is to average the output probability value of multiple base classifiers to obtain the predicted probability value of the final model. Since the classification ability of each base classifier is inconsistent, the classification ability of each base classifier cannot be extracted by using simple averaging directly. Therefore, constructing a model for DR detection based on deep ensemble learning with attention mechanism encounters the following problems.

1. How to handle unbalanced DR dataset and
2. How to set base classifier weight parameters in ensemble learning.

## 2. Related Work

Generally, both dichotomy and multiclassification can be used to classify patients with DR (Pires et al., 2017; Araujo et al., 2020; Porwal et al., 2020; Quellec et al., 2021). The dichotomy method can only determine whether a patient suffers from DR, while the multiclassification method can detect the severity of the disease. Here, we introduce several studies of DR detection algorithms applied with these two classification methods.

Binary classification algorithms of DR are widely applied. Qomariah et al. (2019) proposed to use transfer learning in CNN to extract features, based on which the support vector machine (SVM) algorithm was used to classify DR. Chakrabarty (2018) published a deep learning method for the detection of DR. It first converts pictures into gray-scale, second resizes the pictures to 1000*1000, then scales the pixel values to between 0 and 1, and, finally, inputs the preprocessed picture into CNN to predict the category to which the pictures belong. Chakrabarty and Chatterjee reported an offbeat technique for diabetic retinopathy detection using computer vision (Chakrabarty and Chatterjee, 2019). It first preprocesses pictures, including grayscale conversion, threshold processing, size adjustment, and pixel scaling. Then, the processed images are input into a CNN to extract features, and finally, the features are input into the SVM algorithm for classification. Herliana et al. (2019) applied the particle swarm optimization (PSO) method to select the best DR features, and then the selected features were further classified using the classification method of neural network. Gautam et al. (2019) used MATLAB based image processing to diagnose DR. First, the image is converted to the specified size and then converted to a grayscale image. Then, adaptive histogram equalization is carried out, and the processed image is distinguished with a threshold value. When the pixel value is greater than the threshold value, this part will be changed to white, otherwise, it will be changed to black. Finally, the number of white pixels is counted. After processing several images, the threshold value of the number of white pixels is determined, which is eventually used for category prediction of images. For the classification of retinopathy and non-retinopathy, Roychowdhury et al. (2014) proposed a computer-aided screening system (DREAM) that combines four machine learning algorithms.

The second approach is to classify DR into multiple categories according to the severity of Kanth et al. (2013) proposed applying “gray scale conversion,” “histogram equalization,” “application of digital filters,” “gradient magnetics segmentation,” and “finally fuzzy c clustering” to extract three features including the sum, average, and sum of exudates of the white pixels with a value of “1” in the binary image, and then used the multi-layer perceptron to classify images. Zhang et al. (2021) developed an early fundus abnormality screening system (DeepUWF). Firstly, six image pre-processing techniques were used to enhance the image, and then the image was input into the CNN for image classification. Carrera et al. (2017) first extracted the information of blood vessel, microaneurysm, and hard exudate based on morphology, and then transformed the information into 8 features. Finally, the 8 features were input into the support vector machine for classification. Wu and Hu (2019) used flip, fold, and contrast adjustment to do upsampling; then the pictures are input to the VGG19, Resnet50, and Inception V3 networks which are trained in the ImageNet data set for transfer learning; and finally, the predicted category of the picture is obtained. Jayakumari et al. (2020) firstly normalized the Images and then divided the data set into two categories (normal class and disease class including mild, moderate, severe, and proliferative DR) for model training. Finally, according to the model output probability and the set threshold value, the category described in the picture is judged.

The advantages and disadvantages of the above two methods are summarized in the following Table 1.

**Table 1:** Difference between dichotomy and multiclassification in diabetic retinopathy (DR).

**Method**	**Advantages**	**Disadvantages**
Dichotomy	High accuracy	Prone to under or overtreatment
Multiclassification	Can give doctors more accuracy staging of the disease to get the optimal treatment plan	Small differences in images between different levels of the disease, average accuracy

In this study, we performed multiclassification for the DR dataset. The main contributions of this article are the following two aspects.

1. To deal with the extremely unbalanced DR dataset, the total number of images to be upsampled is divided equally among each image, and upsampling is performed by three transformations, including left-right symmetric transformation, up-down symmetric transformation, and random-angle rotation, to achieve the equalization of the dataset. This way of processing can solve the large classification error of the model caused by the unbalance.
2. The voting weight of each network model in the ensemble learning is set according to the F1 value validated by the base classifier to improve the detection effect.

## 3. Detection Algorithm of DR Based on Deep Ensemble Learning and Attention Mechanism

### 3.1. System Architecture

**Figure 1:**
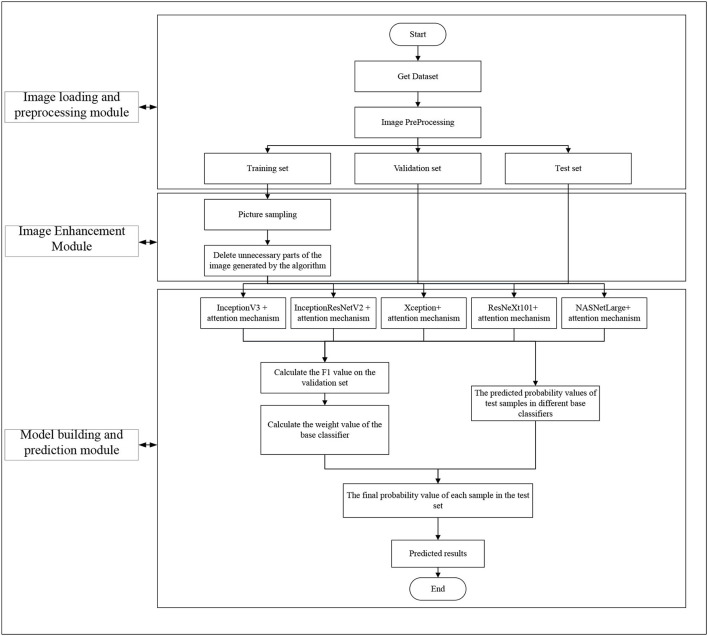
Flow chart of system architecture.

In this study, we constructed a deep ensemble learning algorithm called DR-IIXRN, whose structure is illustrated in Figure 1 and Algorithm 1. The programs of DR-IIXRN can be divided into three modules: image loading and preprocessing, image enhancement, and model building and prediction.

**Algorithm 1:** Experimental structure

Input: *Data* (the dataset), *Base*_*classifier*_*list*=[L1, L2, L3, L4, L5].
Output: Test set prediction category for each test set sample *T* = *T*_1_, *T*_2_, …, *T*_*N*_
1: Algorithm 2 is used to preprocess the image of Dataset *Data* and obtain *Data*_*process*. 2: The *Data*_*process* is divided into the training set *data*_*train*, the validation set *data*_*valid* and the test set *data*_*test* in a 3:1:1 ratio. 3: Algorithm 3 is used to perform image enhancement operation on the training set *data*_*train* image to obtain *data*_*train*_*process*. 4: Algorithm 4 is used to calculate the balanced F score of the base classifier, and *F*1_*list* and *Test*_*pro* are obtained. 5: Calculate the weight value of each base classifier. (1) $$\begin{array}{r} \lambda i=\frac{F1\text{\_}list\left[i\right]}{\left(\sum_{p=1}^{q}F1\text{\_}list\left[p\right]\right)}+\left(F1\text{\_}list\left[i\right]-\frac{\sum_{p=1}^{q}F1\text{\_}list\left[p\right]}{q}\right)*n \end{array}$$ In this formula, q represents the number of base classifiers, n is the parameter that represents the gap between the good and bad algorithms. 6: Calculate the final probability values for each category in the test set sample. (2) $$\begin{array}{r} \left(\sum_{i=1}^{5}\lambda i*Test\text{\_}pro\left[i\right]\right) \end{array}$$ Where i represents each base classifier. 7: Test Set Selection Process In the test, the category with the largest probability value was selected to determine the category *T*_*m*_ of the final test sample, Wherein, m is the sample number of the test set.

**Algorithm 2:** Image preprocessing

**Input**: sample dataset : *Data*.
**Output**: processed dataset : *Data*_*process*.
1: Define the list of stored images after preprocessing:*Data*_*process*=[]. 2: **for** *image*→*Data* **do** 3: Cut pixels in an image where the pixel values in the entire row or column are all below 7. 4: Get the smaller value of image height and image width. 5: The center point of the image is taken as the center of the circle, and the smaller value of height and width is taken as the diameter to determine a circle. All pixel values in the non-circular region are replaced with 0. 6: Cut pixels in an image where the pixel values in the entire row or column are all below 7. 7: Scale Image to 299. 8: Add the Image to the *Data*_*process*. 9: **end for** 10: **return** *Data*_*process*.

**Algorithm 3:** Image enhancement

**Input**: Training dataset: *data*_*train*.
**Output**: processed training dataset: *data*_*train*_*process*.
1: Define the enhanced storage list of images:*data*_*train*_*process*=[]. 2: Get a set of images for each category in *data*_*train*:data_train0, data_train1, data_train2, data_train3, data_train4. 3: *data*_*train*_*list*=(data_train1, data_train2, data_train3, data_train4). 4: **for** *data*_*train*_*i*→*data*_*train*_*list* **do** 5: Calculate the difference between the sample sizes of category *data*_*train*_*i* and category *data*_*train*0 (normal sample): *numSub*. 6: According to *data*_*train*_*i* and *numSub*, calculate the number of images to be upsampled for each image:*numAdd*. 7: **for** *image*→*data*_*train*_*i* **do** 8: Left-right symmetrical transformation, up-down symmetric transformation, and random-angle rotation transformation on image to stack *numAdd* image. 9: Add the stack image to the *data*_*train*_*process*. 10: **end for** 11: **end for** 12: **return** *data*_*train*_*process*.

**Algorithm 4:** Calculate the base classifier F1 value

**Input**: Base classifier list:*Base*_*classifier*_*list*, training set: *data*_*train*_*process*, validation set:*data*_*valid*, test set: *data*_*test*.
**Output**: Base classifier F1 value: *F*1_*list*,the initial prediction probability value of the test set sample:*Test*_*pro*.
1: Defines a list of base classifier F1 values: *F*1_*list*=[]. 2: Define a list of base classifier models:*Model*_*list*=[]. 3: Define the list of initial probability values for the test set sample:*Test*_*pro*=[]. 4: **for** *base*_*classifier*→*Base*_*classifier*_*list* **do** 5: Remove the top layer in base_classifier, load the weight parameters of the corresponding model in “imageNet,” and get the model:base_model. 6: Add the CBAM attention mechanism module after base_model. 7: Add the classification model output layers module after base_model. Get the model:model. 8: The training set *data*_*train*_*process* and the validation set *data*_*valid* are trained in the model. 9: Remove the limitation in base_model that the training parameters remain the same, and re-train again. The model is added to *Model*_*list* at the end of the training. 10: **end for** 11: **for** *model*→*Model*_*list* **do** 12: The validation set *data*_*valid* is tested in the model to get the F1_score, and the result is added to the *F*1_*list*. 13: The test set *data*_*test* is tested in the model to get the category prediction probability value, which is added to *Test*_*pro*. 14: **end for** 15: **return** *F*1_*liste, Test*_*pro*.

### 3.2. Dataset

**Figure 2:**
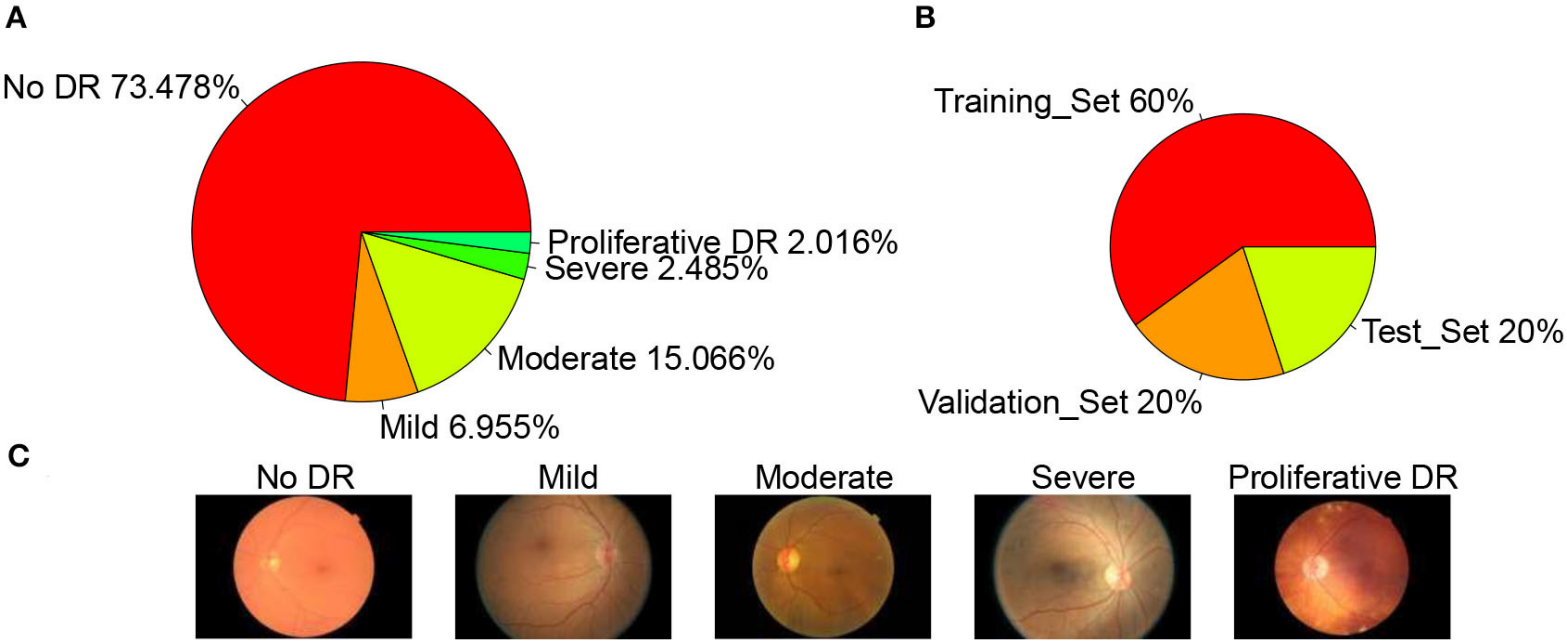
Datasets. **(A)** Datasets sample distribution, **(B)** Set sample distribution, and **(C)** Example of each category.

The dataset used in this article is from the Diabetic Retinopathy Detection Competition in the Data Modeling and Data Analysis Competition Platform (Kaggle:https://www.kaggle.com/c/diabetic-retinopathy-detection/data). A total of 35,126 image samples were collected and classified into five categories: normal, mild, moderate, severe, and proliferative DR. The sample size of each category was 25,810, 2,443, 5,292, 873, and 708, respectively. The sample distribution is shown in Figure 2A. A typical example of each category is shown in Figure 2C. The data set is divided into a training set, validation set, and test set according to the ratio of 3:1:1, so the sample size of each set is 21,074, 7,026, and 7,026, respectively. The sample distribution is shown in Figure 2B below.

### 3.3. Image Preprocessing

**Figure 3:**
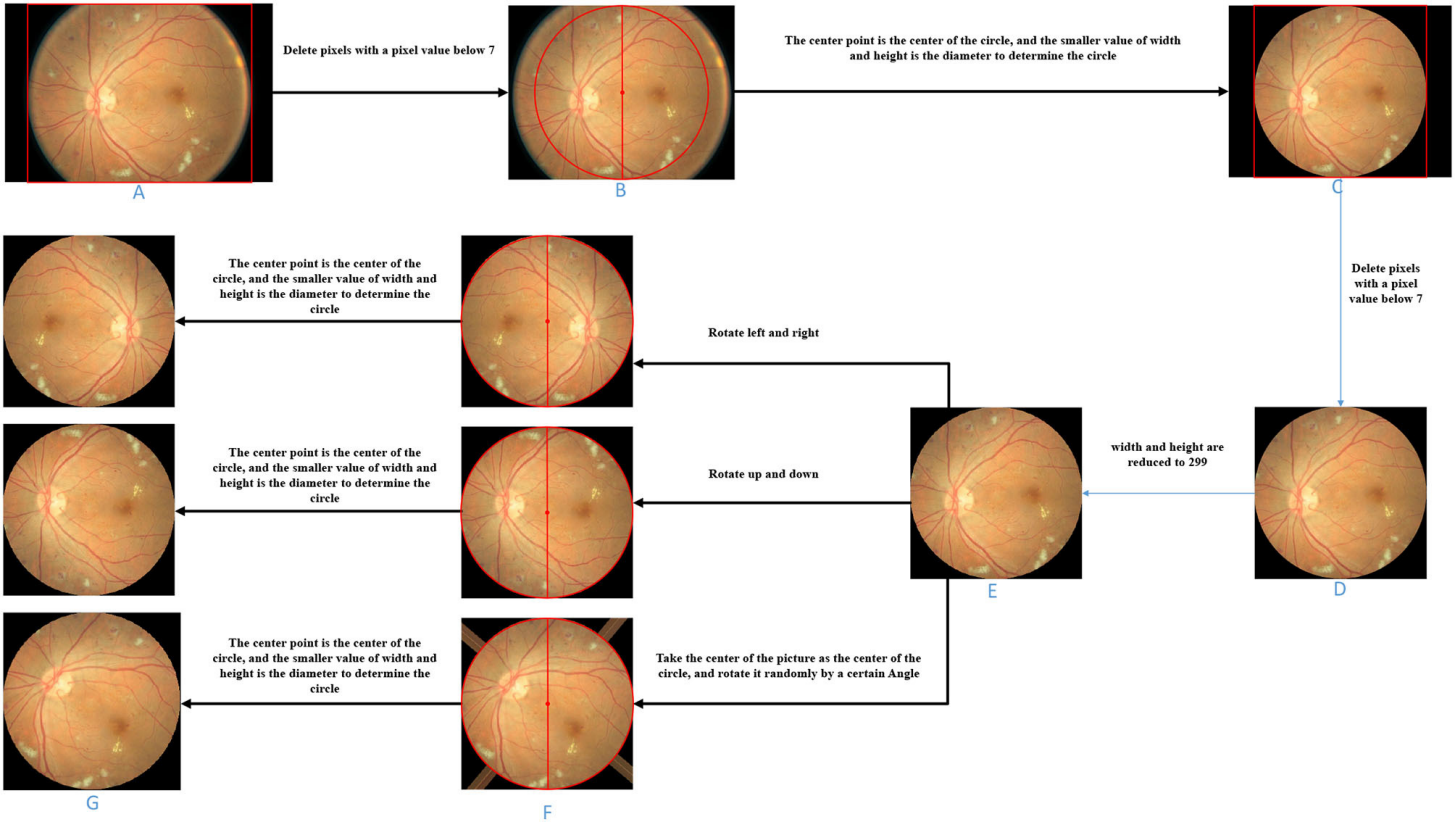
Image processing schematic diagram of each stage. **(A)** Original fundus image. **(B)** Image obtained by deleting entire rows and columns of pixels with a pixel value less than 7. **(C)** Take the center point of B as the center of the circle, and the smaller value between the height and the width as the diameter to determine the circle and then fill the area outside the circle with pixel values as 0. **(D)** On the basis of C, the image obtained after deleting entire rows and columns of pixels with a pixel value less than 7. **(E)** Zooms the image to the specified size. **(F)** Image obtained by three image transformations on the basis of E. **(G)** Take the center point of F as the center of the circle, and the smaller value between the height and the width as the diameter to determine the circle and then fill the area outside the circle with pixel values as 0.

The image preprocessing is demonstrated in Figure 3.

1. Cut pixels in an image where the pixel values in the entire row or column are all below 7. Pixels with pixel values below 7 are all black, which does not help the subsequent data analysis. In general, removing pixels with lower pixel values in the image can reduce the time complexity of the network model. This step can remove the non-eye region as much as possible and increase the robustness of the algorithm. Therefore, as shown in Figure 3, we delete the pixel rows and columns in which pixel values are all lower than 7 in the picture (Figure 3A) to get the picture (Figure 3B).
2. Determine a circle by taking the image center as the center and the smaller value between the image height and width as the diameter. Fill the area outside the circle with pixel values as 0 to obtain the image with the circular eye area retained. Based on the roughly circular nature of fundus images, we keep only the circular part of the image and remove redundant information that is not helpful for diabetic retinal classification, which can both improve the accuracy of the classification model (network model or base classifier) and reduce the time complexity. Referring to Figure 3, we take the center point of Figure 3B as the center of the circle, and the smaller value between the height and the width as the diameter to determine the circle and then fill the area outside the circle with pixel values as 0 and get the picture (Figure 3C).
3. For the image with the circular eye area, delete the pixels in an image where the pixel values in an entire row or column is all below 7 again and scale the image to 299*299px. Since the radius of the circle is determined by the smaller value of the width and height of the image in step (2), there may be some “eyeball” pixels that are not included in the determined circle and are filled with a pixel value of 0. Therefore, in this step, it is necessary to cut the pixels where the pixel values in the entire row or column are all below 7 again. As shown in Figure 3, we delete the pixel rows and columns in which pixel values are all lower than 7 in the picture (Figure 3C) to get the picture (Figure 3D). Further, both the width and height of the image (Figure 3D) are scaled to 299 px to get the image (Figure 3E).

### 3.4. Image Enhancement

#### 3.4.1. Picture Sampling

First, the number of pictures that need to be upsampled for each picture in each diseased class (class 1, 2, 3, 4) was calculated according to the following formula.


(3) $$\begin{array}{r} Add_{i}=\frac{\left(N_{0}-N_{i}\right)}{N_{i}}\;i=1,2,3,4 \end{array}$$

Where *Add*_*i*_ is the number of images to be added per image in class i, *N*_0_ is the number of pictures of class 0 in the training set, and *N*_*i*_ is the number of pictures of class i in the training set.

Second, random-upsampling was performed to increase the number of images in each class to obtain a balanced dataset. The upsampling numbers for each category are listed in Table 2 below.

**Table 2:** Upsampling image number information.

**Category**	**Number of**	**Number of images**	**Number of images**
	**original images**	**added per picture**	**after upsampling**
0	15,472	0	15,472
1	1,445	9	14,450
2	3,183	3	12,732
3	553	26	14,931
4	421	35	15,156

The image transformation methods in upsampling include left-right symmetrical transformation, up-down symmetric transformation, and random-angle rotation transformation. The left-right symmetric transformation refers to the mirror image inversion with the vertical centerline of the image as the axis of symmetry. The up-down symmetric transformation refers to the image inversion with the horizontal centerline of the picture as the axis of symmetry. The random-angle rotation transformation means rotating an image with a random angle with the center of the picture as the center of the circle. As shown in Figure 3, three sampled pictures (Figure 3F) can be obtained from picture (Figure 3E) by the three transformation methods.

#### 3.4.2. Deletion of Unnecessary Areas

During the random-angle rotation transformation, some pixels outside the “eyeball” area may be filled with pixel values higher than 7. As shown in the third picture in Figure 3F, bright bars near the four corners appear. Therefore, this step is to remove these bright pixels. For each upsampled image, determine a circle with the center of the image as the center of the circle and the half of the side length as the radius and then fill the area outside the circle with pixel values as 0. As shown in Figure 3, unnecessary areas of the three sampling images in Figure 3F were removed and three upsampling images (Figure 3G) with circular eye areas were obtained.

### 3.5. Introduction to Network Models

We used five top-ranked and widely used architectures trained on the ImageNet Large-scale Visual Recognition Challenge (ILSVRC): Inception V3, InceptionResNet V2, Xception, ResNext101, and NasnetLarge.

#### 3.5.1. Inception V3 Network Model

Since Yann LeCun, the father of CNN, built LeNet5 (LeCun et al., 1998), which started the research boom of CNN, many scholars have been working hard in this field. In the 2014 ImageNet Competition, the GoogLeNet (Inception V1) (Szegedy et al., 2015) network proposed by Szegedy and Liu et al. and the VGGNet (Simonyan and Zisserman, 2015) network proposed by Simonyan and Zisserman won the first and second place, respectively. The GoogLeNet with a network depth of 22 layers is much smaller than VGGNet both in terms of network parameters and network size. Inception V2 (Ioffe and Szegedy, 2015) is a network model optimized on the basis of GoogLeNet, and Batch-Normalization (BN) is added to the network model, which accelerates the training speed of the model. Inception V3 (Szegedy et al., 2016) is similar to Inception V2, except that “factorization into small cons” is introduced on the basis of Inception V2, which means any n*n convolution kernel can be disassembled into a combination of size 1*n and n*1, and this operation allows the number of parameters to be greatly reduced. Since the introduction of the Inceptionv3 network, it has been used by a large number of researchers (Dongmei et al., 2020; Li W. et al., 2020). Dong et al. (2020) proposed a network framework. The network will effectively solve the complexity and individual differences of the cervical cell texture. Combined with the feature of Inception v3 and artificial cell classification algorithm, effectively improve the cervical cells recognition accuracy. Liu et al. (2020) proposed a classification algorithm based on improved InceptionV3 while Center loss CNN is proposed to improve the accuracy of obscured targets.

#### 3.5.2. InceptionResNet V2 Network Model

InceptionResNet V2 (Szegedy et al., 2017) is a deep network model proposed by Szegedy et al. Based on Inception, ResNet (He et al., 2016) is introduced to add shallow features to higher-level features through another branch for the purpose of feature reuse and also to avoid the gradient disappearance problem of deep networks. Since the introduction of InceptionResNet V2 network, it has been used by a large number of researchers (Kamble et al., 2019; Peng et al., 2020). Ferreira et al. (2018) proposes a deep neural network method for classification of breast cancer histology images using InceptionResnet V2 transfer learning. First, the added top layer is trained and some of the previously frozen feature extraction layers are fine-tuned a second time. (Thomas et al., 2020) proposes the use of Inception- resnet-V2 as feature extraction and the extracted features are fed into two different classifier support vector machines and random forest to classify vehicle types.

#### 3.5.3. Xception Network Model

Xception (Chollet, 2017), a deep network model proposed by Francois Chollet et al. in 2017, is an improved version of Inception V3. It mainly uses depth-wise separable convolution to replace the convolution operation in the original Inception V3, which can improve the performance of the network model to some extent. Since the introduction of the Xception network, it has been used by a large number of researchers (Rismiyati et al., 2020; Wu et al., 2020). Farag et al. (2021) proposed the use of residual network and Xception network for COVID-19 diagnosis, and the results show that the use of random search-optimized residual network and Xception network can achieve good classification results. Yao et al. (2021) propose an improved Xception network, in which L2 norm and mean regularization are added to the original Xception network, and the classification indicators of the tuned Xception network are greatly improved.

#### 3.5.4. ResNeXt101 Network Model

ResNeXt101 is a deep network model put forward by Xie et al. (2017). The study suggests that increasing cardinality is more effective than increasing depth and width, which improves the accuracy of the model without obviously increasing the order of magnitude of parameters, and also allows it to have fewer hyperparameters, which facilitates model portability. Since the introduction of ResNeXt network, it has been used by a large number of researchers (Koné and Boulmane, 2018; Pant et al., 2020). Go et al. (2020) proposes a visualization-based malware analysis method. First, the properties of binary executable files of original malware are converted into grayscale images, and ResNeXt network is used to classify grayscale images to realize malware analysis. Cao et al. (2021) proposed ResNeXt as a backbone network with few parameters and high accuracy, and then used k-means++ clustering algorithm to get an anchor box that was closer to the real box, which helped the model to carry out regression detection. The accuracy of the improved model was significantly improved.

#### 3.5.5. NASNetLarge Network Model

NASNetLarge is a deep network model proposed by Zoph et al. (2018). NASNetLarge can automatically generate network structures without the need to design the network model manually. This model can greatly reduce the number of parameters while ensuring accuracy. Since the introduction of NASNet network, it has been used by a large number of researchers (Ahmed et al., 2020a; Bharati et al., 2021). Bakkali et al. (2020) proposes a cross-mode deep network, which can capture text content and visual information contained in document images. Image features use NASNetLarge network, text feature extraction uses Bert network, and the cross-mode deep network greatly improves classification indicators. Ahmed et al. (2020b) proposed to use a framework combining three pre training networks (Xception, Inception-ResNet-V2, and NasNetLarge) and Error Correcting Output Codes (ECOC) to solve the classification of skin damage.

### 3.6. Attention Mechanism

**Figure 4:**
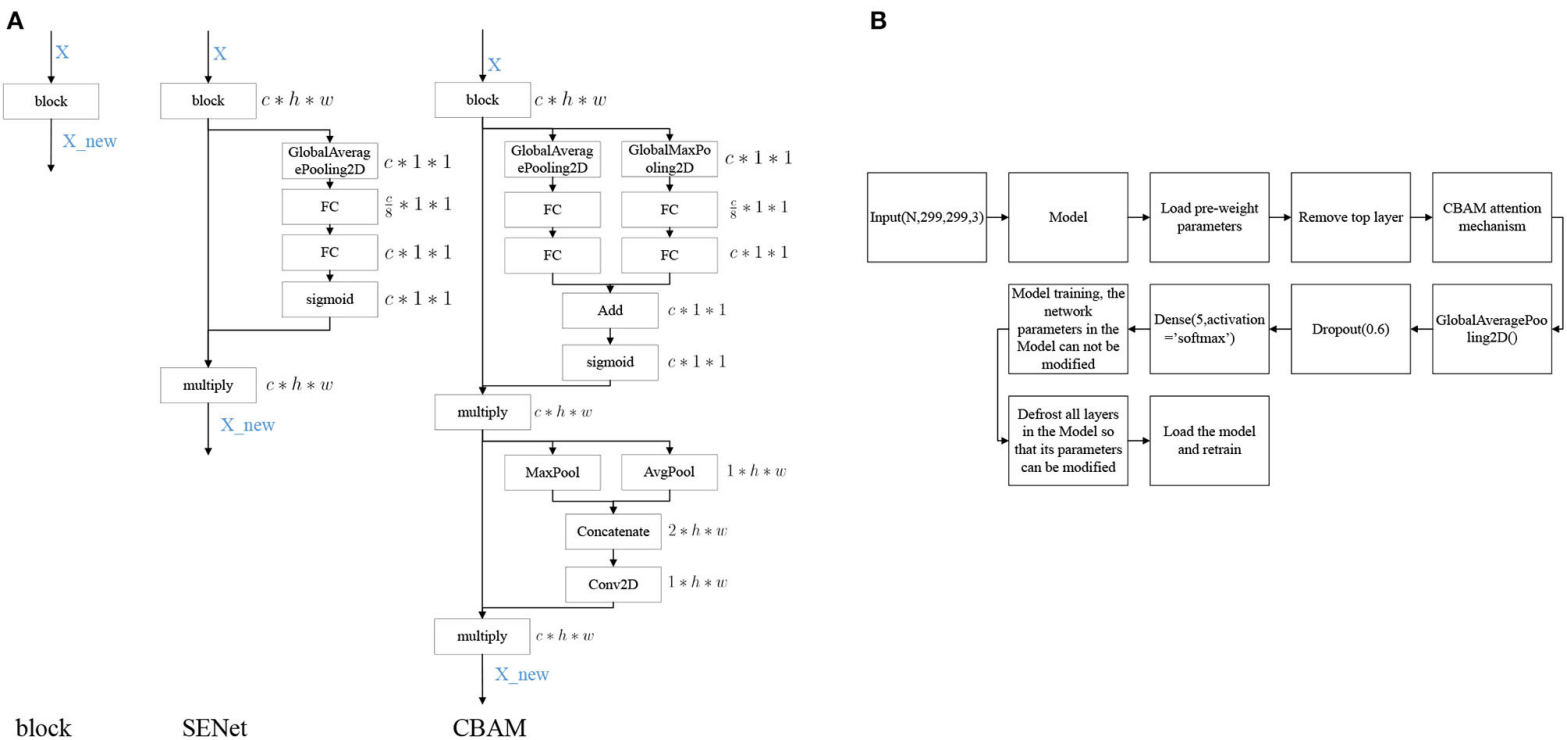
Model modification process. **(A)** Diagram of attention mechanism network structure, **(B)** Model training process.

Convolutional Block Attention Module (CBAM) (Woo et al., 2018) denotes the attention mechanism module of the convolutional module, which is an attention mechanism module that combines spatial and channel dimensions and can achieve better results compared to Squeeze-and-Excitation Networks (SENet) (Hu et al., 2020), an attention mechanism that focuses only on the channel dimension. The network structures of SENet and CBAM added into the block are shown in Figure 4A.

### 3.7. Model Building

The modeling stages are descripted in Figure 4B. The models are mainly composed of Inception V3, InceptionResNet V2, Xception, ResNeXt101, and NASNetLarge. On the basis of these models, the top layer is removed, and the CBAM attention mechanism and model output layers are added. After the model is constructed, transfer learning is performed, keeping the parameters in the network model immutable and only modifying the parameters of the attention mechanism and the output layers module. Once the model is trained, the parameters of the network model are free to be modified, and the training is performed again to obtain a suitable network model for DR.

## 4. Experiment

### 4.1. Experimental Conditions

The experimental environment is Linux x86_64, NVIDIA Tesla V100, and 16GB memory. This experiment is based on Python version 3.7.9, TensorFlow version 2.3.0, and Keras version 2.4.3.

### 4.2. Evaluation Criteria

To evaluate the performance of the model, the accuracy, recall, precision, and F1-score were calculated.

The combinations of predicted outcomes of the classifier and true categories of samples were classified as True Positive (TP), True Negative (TN), False Positive (FP), and False Negative (FN). TP means that the classification model predicts positive samples as positive samples. TN means that the classification model predicts negative samples as negative samples. FP means that the classification model predicts negative samples as positive samples. FN means that the classification model predicts positive samples as negative samples.


(4) $$\begin{array}{r} Accuracy=\frac{\left(TP+TN\right)}{TP+TN+FP+FN} \end{array}$$

(5) $$\begin{array}{r} Precision=\frac{\left(TP\right)}{TP+FP} \end{array}$$

(6) $$\begin{array}{r} Recall=\frac{\left(TP\right)}{TP+FN} \end{array}$$

(7) $$\begin{array}{r} F1-Score\left(F1\right)=\frac{\left(2*Precision*Recall\right)}{Precision+Recall} \end{array}$$

(8) $$\begin{array}{r} Specificity=\frac{\left(TN\right)}{\left(FP+TN\right)} \end{array}$$

“Accuracy” represents the proportion of all correct judgments of the classifier to the total number of observations. “Precision” is the proportion of subjects correctly predicted to be positive among all positive predictions. “Recall” is a ratio of correct positive predictions to the overall number of positive instances in the dataset. “Specificity” is the proportion of negative instances identified to all negative instances. “F1” is the harmonic mean of precision and recall. AUC is a performance measure for classification problems under various threshold Settings. F1 values range from 0 to 1, and the best value is 1.0, and the worst value is 0.0.

### 4.3. Selection of Experimental Settings

In order to investigate the potential of different experimental settings in the image processing module and the model building module in the proposed framework, we conducted experiments for the following different cases and compared the performance of the model under each case. All experiments applied Inception V3 network and CBAM attention mechanism to construct classification models. A different case of the experimental results is shown in Figure 6A and Table 3. According to the test results, the classification index using Case F reached the highest level, and this process was also identified as the data preprocessing scheme in this article.

1. Case A: No pre-processing steps (using raw data).
2. Case B: On the basis of A, weight parameters are added during model classification.
3. Case C: On the basis of A, the images in the training set are upsampled using horizontal translation, vertical translation, horizontal rotation, vertical rotation, and random angle rotation.
4. Case D: On the basis of A, the images in the training set are upsampled using horizontal rotation, vertical rotation, and random angle rotation.
5. Case E: On the basis of D, the Inception V3 containing network parameters from the competition of “ImageNet” is used as the network model. Here, the network parameters of Inception V3 are not modifiable, distinguishing from the random network parameters used in Case A, B, C, and D.
6. Case F: On the basis of E, the fine-tuning technique is applied.

**Table 3:** The influence of different cases on evaluation indexes.

**Case**	**Label**	**Precision**	**Recall**	**F1-score**	**Support**
A	No DR	0.74	1	0.85	5,175
	Mild DR	0	0	0	493
	Moderate DR	0	0	0	1,049
	Severe DR	0	0	0	160
	PDR	0	0	0	149
B	No DR	0.79	0.23	0.36	5,175
	Mild DR	0.08	0.46	0.14	493
	Moderate DR	0.14	0.01	0.02	1,049
	Severe DR	0.03	0.19	0.06	160
	PDR	0.05	0.62	0.1	149
C	No DR	0.75	0.97	0.85	5,175
	Mild DR	0.11	0.01	0.02	493
	Moderate DR	0.33	0.06	0.11	1,049
	Severe DR	0.26	0.12	0.17	160
	PDR	0.26	0.1	0.14	149
D	No DR	0.8	0.94	0.86	5,175
	Mild DR	0.11	0.02	0.03	493
	Moderate DR	0.43	0.28	0.34	1,049
	Severe DR	0.4	0.23	0.29	160
	PDR	0.53	0.21	0.3	149
E	No DR	0.83	0.92	0.87	5,175
	Mild DR	0.1	0.04	0.05	493
	Moderate DR	0.52	0.45	0.48	1,049
	Severe DR	0.46	0.32	0.37	160
	PDR	0.71	0.5	0.59	149
F	No DR	0.83	0.92	0.87	5,175
	Mild DR	0.1	0.04	0.05	493
	Moderate DR	0.53	0.44	0.48	1,049
	Severe DR	0.46	0.33	0.38	160
	PDR	0.69	0.5	0.58	149

In Case A, the original dataset is unbalanced and there are far more normal samples than other types of samples in the dataset, so the model prediction results are completely biased to the side of normal samples and the model does not play any role in sample classification. To deal with the imbalanced data, we tested the following methods in Cases B, C, and D.

In Case B, a penalty for prediction errors in categories with small sample sizes is added to the model, and the weight parameters for each category are calculated as follows:


(9) $$\begin{array}{r} Weight=\frac{n\text{\_}samples}{n\text{\_}classes*bincount\left(y\right)} \end{array}$$

Where *n*_*samples* represent the total number of picture samples, *n*_*classes* represent the number of categories, and *bincount*(*y*) represents the sample size of each category in the training set. Weight is the weight corresponding to each category. The lower the sample size of the category, the higher its weight. Comparing the results to Case A, we can see that the detection ability for the categories with smaller sample sizes has been slightly improved.

In Case C, we performed horizontal translation, vertical translation, horizontal rotation, vertical rotation, and random angle rotation to carry out the upsampling of the images. The sample size was made approximately the same in each category by up-sampling. Compared with Case B, Case C showed a certain improvement in the detection of late-stage DR, but showed a certain decrease for category 1. However, compared with Case B, the improvement effect for other categories was significant. Therefore, Case C showed an overall improvement compared with Case B.

**Figure 5:**
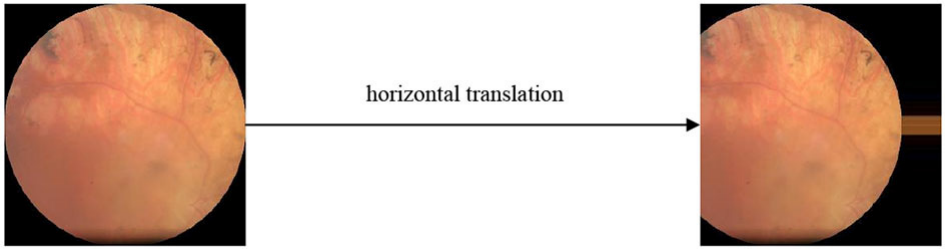
Horizontal translations.

Since the two transformations, horizontal translation and vertical translation, produce images that differ too much from the images of the actual samples, as shown in Figure 5, and may affect the classification effect, we removed these two types of upsampling in Case D. The classification effect for categories with the small sample size is significantly improved in Case D.

However, in Case D, we started from scratch to solve the weight parameters of the model. Due to the small sample size, the training for the complex network model was not sufficient. Case E uses the network parameters in the “ImageNet” competition, which is calculated by a large amount of data. The network parameters are very suitable for the model. Therefore, the effect in Case E is improved to some extent compared with that in Case D.

In Case F, compared with Case E, the weighted parameters in Inception V3 were set to be modifiable in training. We fine-tuned parameters so that the model can be suitable for the detection of DR. Therefore, Case F achieved an improvement over Case E.

### 4.4. Weighted Voting Model

Ensemble learning combines diverse models (henceforth classifiers) to obtain better predictive performance.

This article uses the weighted voting method mainly through the following steps.

1. First, get the F1_score of each base classifier on the validation set. Test the test set in the model, and get the prediction probability value Test_pro of the test set in each category.
2. Calculate the weight value of each base classifier through the following formula. Wherein, F1_list is the F1 score of each base classifier in the verification set, q is the number of base classifiers, so $\frac{F1\text{\_}list\left[i\right]}{\sum_{p=1}^{q}F1\text{\_}list\left[p\right]}$ represents the percentage of F1 score of each base classifier in the total score, and $\frac{\sum_{p=1}^{q}F1_{l}ist\left[p\right]}{q}$ represents the mean of F1 of each base classifier. $F1\text{\_}list\left[i\right]-\frac{\sum_{p=1}^{q}F1\text{\_}list\left[p\right]}{q}$ represents the difference between each base classifier and the mean, so the n value is to amplify the difference between the base classifiers.
  (10) $$\begin{array}{r} \lambda i=\frac{F1\text{\_}list\left[i\right]}{\sum_{p=1}^{q}F1\text{\_}list\left[p\right]}+\left(F1\text{\_}list\left[i\right]-\frac{\sum_{p=1}^{q}F1\text{\_}list\left[p\right]}{q}\right)*n \end{array}$$
3. Calculate the probability of each sample in the test set.
  (11) $$\begin{array}{r} T\left[i\right]=\lambda i*Test\text{\_}pro\left[i\right] \end{array}$$
4. After obtaining the predicted probability value of each sample in each category, the category with the largest probability value is the predicted category value.

**Figure 6:**
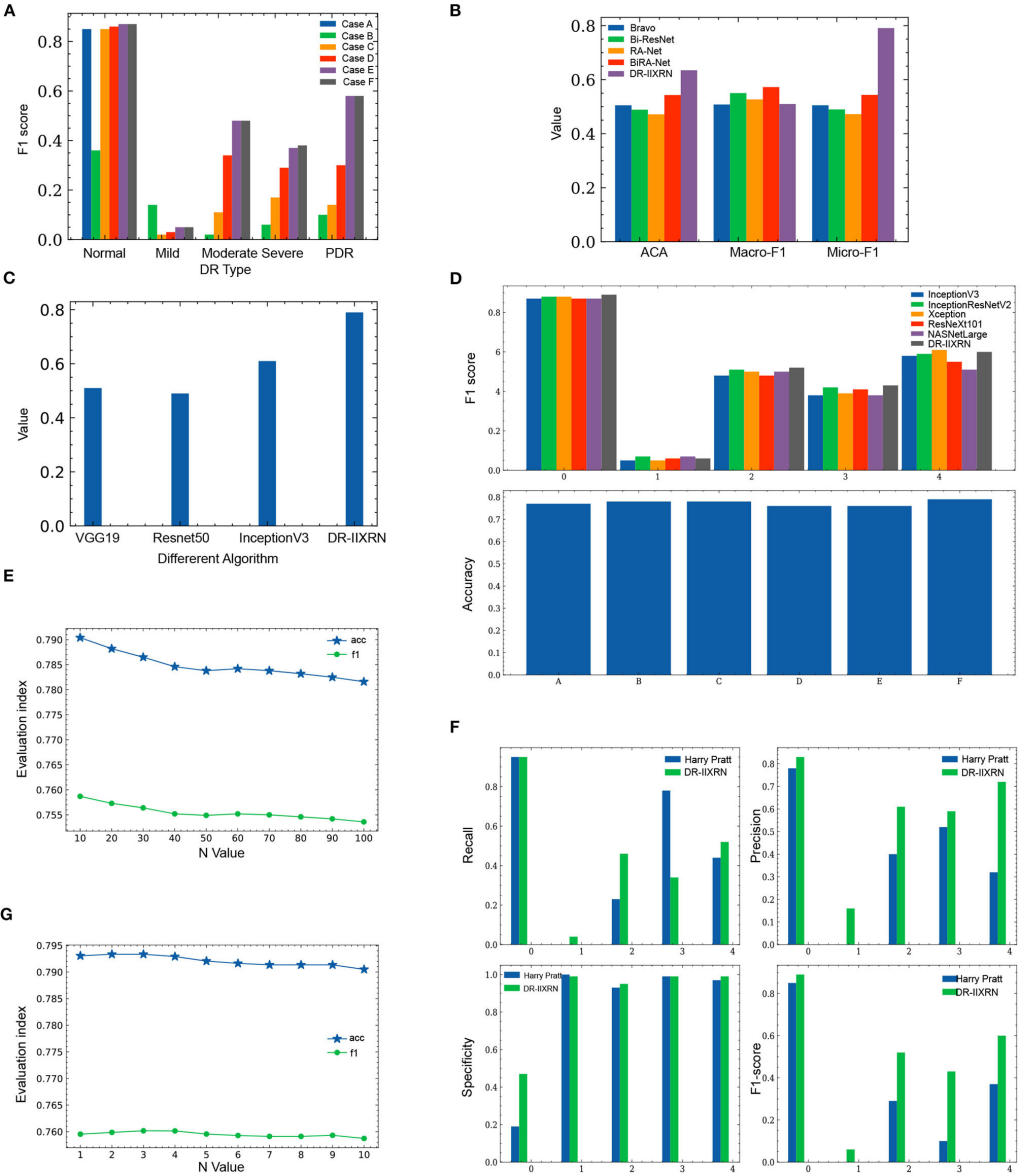
Comparison of experimental results. **(A)** Influence of different image preprocessing schemes on F1 value. **(B)** Comparison of the performance of DR-IIXRN with Zhao and Bravo proposed algorithms. **(C)** Compared the performance of DR-IIXRN with Wu proposed algorithms. **(D)** Comparison of the performance of DR-IIXRN with the five base classifiers. **(E)** Influence of n value in formula 10 between 10 and 100 on evaluation index. **(F)** Comparison of the performance of DR-IIXRN with Pratt proposed algorithms. **(G)** Influence of n value in formula 10 between 1 and 10 on evaluation index.

In step 2, the range of n that increases the difference between the base classifiers is generally small. If the data is too large, it will cause the predicted probability values to be exactly the same. We test n values from 10 to 100. Select the most appropriate n to increase the difference between the base classifiers. In the first step of the experiment, n is in the range from 10 to 100. The specific values are 10, 20, 30, 40, 50, 60, 70, 80, 90, and 100. The experimental results are shown in Figure 6E and Table 4.

**Table 4:** The influence of different values of *n* on the evaluation indexes.

**Step**	***N* value**	**Accuracy**	**F1_score**
First step	10	0.7904	0.7587
	20	0.7882	0.7573
	30	0.7865	0.7564
	40	0.7846	0.7552
	50	0.7838	0.7549
	60	0.7842	0.7552
	70	0.7838	0.755
	80	0.7832	0.7546
	90	0.7825	0.7542
	100	0.7816	0.7536
Second step	1	0.7931	0.7595
	2	0.7933	0.7599
	3	0.7933	0.7602
	4	0.7929	0.7601
	5	0.7921	0.7596
	6	0.7916	0.7593
	7	0.7913	0.7591
	8	0.7913	0.7591
	9	0.7913	0.7593
	10	0.7905	0.7587

Figure 6E shows that the model evaluation indexes with n values between 10-100 show a downward trend, mainly because the large n value leads to the consistency of the prediction indexes of most samples when the model predicts samples. So the optimal n is between 1 and 10. In the second step of the experiment, n is in the range from 1 to 10. The specific values are 1, 2, 3, 4, 5, 6, 7, 8, 9, and 10. The experimental results are shown in Figure 6G and Table 4.

Figure 6G shows that when then value is between 1 and 3, the evaluation index of the model shows an overall upward trend. Increasing n value within a certain range can increase the difference between various base classifiers, thus improving the classification ability of the model. Between 3 and 10, the overall model evaluation index shows a downward trend. As can be seen from Formula 10, when n value is too large, the model will keep the predicted values of most samples consistent. Therefore, the optimal n value is 3, which can widen the gap between each base classifier and make the effect of deep ensemble learning reach the optimal level.

We compared the performance of the DR-IIXRN algorithm with the five base classifiers, as shown in Figure 6D and Table 5. In Figure 6D, the horizontal coordinates “0,” “1,” “2,” “3,” and “4” represent “No DR,” “Mild DR,” “Moderate DR,” “Severe DR,” and “PDR,” respectively; “A,” “B,” “C,” “D,” “E,” and “F” represent “Inception V3,” “InceptionResNet V2,” “Xception,” “ResNeXt101,” “NASNetLarge,” and “DR-IIXRN,” respectively. The results show that compared with the basic classifier, deep ensemble learning can effectively compensate for the errors of each base classifier and improve the overall accuracy and F1_score of the classifier.

**Table 5:** Deep ensemble learning vs. base classifiers.

**Case**	**Accuracy**	**Label**	**F1-score**
Inception V3	0.77	No DR	0.87
		Mild DR	0.05
		Moderate DR	0.48
		Severe DR	0.38
		PDR	0.58
InceptionResNet V2	0.78	No DR	0.88
		Mild DR	0.07
		Moderate DR	0.51
		Severe DR	0.42
		PDR	0.59
Xception	0.78	No DR	0.88
		Mild DR	0.05
		Moderate DR	0.5
		Severe DR	0.39
		PDR	0.61
ResNeXt101	0.76	No DR	0.87
		Mild DR	0.06
		Moderate DR	0.48
		Severe DR	0.41
		PDR	0.55
NASNetLarge	0.76	No DR	0.87
		Mild DR	0.07
		Moderate DR	0.5
		Severe DR	0.38
		PDR	0.51
DR-IIXRN	0.79	No DR	0.89
		Mild DR	0.06
		Moderate DR	0.52
		Severe DR	0.43
		PDR	0.6

### 4.5. Comparison of the DR-IIXRN Algorithm and Other Classification Algorithms

In order to verify the performance of the DR-IIXRN algorithm, we collected and downloaded several DR detectors applied in the literature published in the last 5 years.

Firstly, the DR-IIXRN algorithm was compared with the algorithm proposed by Harry Pratt (Pratt et al., 2016). Due to the serious imbalance of the data set, the algorithm in this paper and Harry Pratt both perform poorly in the F1 value of the two categories Mild DR and Severe. However, compared with the algorithm proposed by Harry Pratt, in this article, the effect of data imbalance on category Mild DR and category Severe can be greatly reduced by the image up sampling module. By comparing the metrics of recall, precision, specificity, and F1 value, it can be seen from Table 6, Figure 6F that the detection capability of DR-IIXRN algorithm has obvious superiority. In Figure 6F, the horizontal coordinates “0,” “1,” “2,” “3,” and “4” stand for “No DR,” “Mild DR,” “Moderate DR,” “Severe DR,” and “PDR,” respectively.

**Table 6:** DR-IIXRN vs. Harry Pratt proposed model.

**DR type**	**Recall**		**Precision**		**Specificity**		**F1_score**	
	**DR-**	**Harry**	**DR-**	**Harry**	**DR-**	**Harry**	**DR-**	**Harry**
	**IIXRN**	**Pratt**	**IIXRN**	**Pratt**	**IIXRN**	**Pratt**	**IIXRN**	**Pratt**
No DR	0.95	0.95	0.83	0.78	0.47	0.19	0.89	0.85
Mild DR	0.04	0	0.16	0	0.99	1	0.06	0
Moderate DR	0.46	0.23	0.61	0.4	0.95	0.93	0.52	0.29
Severe DR	0.34	0.78	0.59	0.52	0.99	0.99	0.43	0.1
PDR	0.52	0.44	0.72	0.32	0.99	0.97	0.6	0.37

Next, we conducted experiments with algorithms proposed by Bravo (Bravo and Arbelaez, 2017) and Ziyuan Zhao (Zhao et al., 2019) for the same test set as DR-IIXRN. We evaluated and compared these algorithms by three metrics: average of classification accuracy (ACA), macro-averaged F1 (Macro-F1), and Micro-averaged F1 (Micro-F1). The classification confusion matrix is normalized, and then the average value of the diagonal line is calculated to obtain the average value of the classification accuracy(ACA). Macro-F1 and Micro-F1 were used to evaluate the results of multiple classifications. Macro-F1 and Micro-F1 are computed as simple arithmetic means of per-class F1-scores. The Macro F1-score is defined as the mean of class-wise F1-scores, and Micro F1-score is defined as the harmonic mean of the precision and recall.

Compared with the network architecture proposed by Bi-ResNet and Bravo, this article uses network structures with excellent results from the “ImageNet” competition Inception V3, InceptionResNet V2, Xception, ResNext101, and NASNetLarge as part of the DR-IIXRN, and fine-tune each network structure. This fine-tuning operation can greatly reduce the model training time on the premise of ensuring the accuracy (Kermany et al., 2018). The experimental results are shown in Table 7 and Figure 6B. The DR-IIXRN algorithm showed good performance on ACA and Micro-F1.

**Table 7:** The influence of different algorithms on the evaluation index.

**Algorithm**	**ACA**	**Macro-F1**	**Micro-F1**	**Accuracy**
Bravo	0.5051	0.5081	0.5052	–
Bi-ResNet [Ziyuan Zhao]	0.4889	0.5503	0.4897	–
RA-Net [Ziyuan Zhao]	0.4717	0.5268	0.4724	–
BiRA-Net [Ziyuan Zhao]	0.5431	0.5725	0.5436	–
VGG19 [Yuchen Wu]	–	–	–	0.51
Resnet50 [Yuchen Wu]	–	–	–	0.49
InceptionV3 [Yuchen Wu]	–	–	–	0.61
DR-IIXRN	0.6347	0.51	0.791	0.79

Finally, we compared the DR-IIXRN algorithm with the algorithms proposed by Yuchen Wu (Wu and Hu, 2019) on accuracy. In this article, we use five commonly used network models at the stage of model building and integrate the output results of various models, which can effectively compensate for the error of each base classifier. Therefore, compared with the model proposed by Yuchen Wu, this article can improve the detection ability of DR to a certain extent. It can be seen from Figure 6C and Table 7 that the DR-IIXRN algorithm greatly improves the classification ability of DR samples.

### 4.6. Experimental Expansion

**Figure 7:**
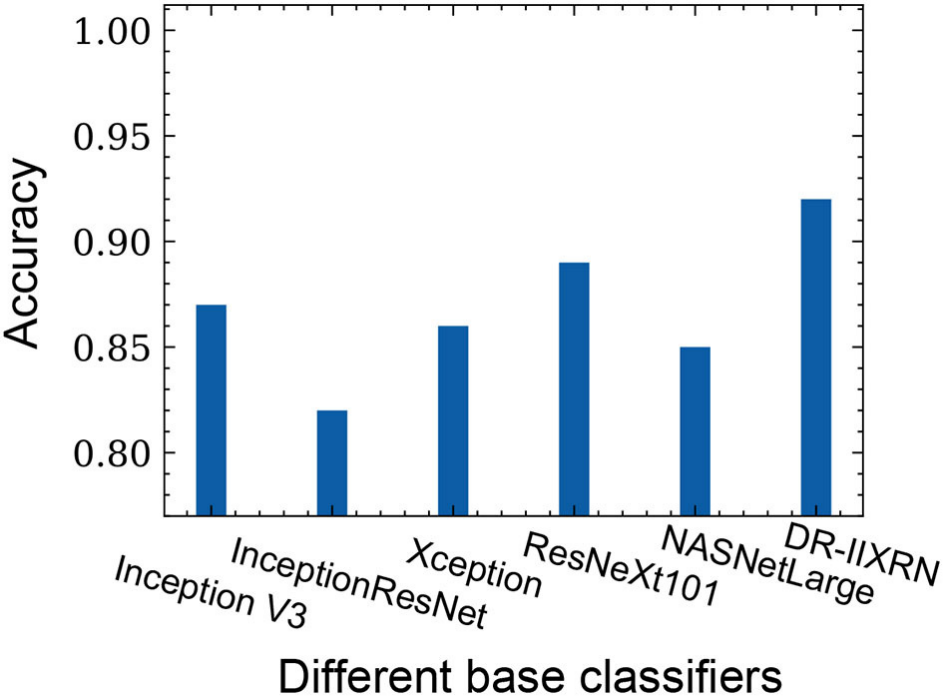
The influence of different base classifiers on accuracy.

In this study, 945 fundus images were collected from Beijing Chaoyang Hospital, Capital Medical University. The categories of images are shown in the following Table 8. In this article, 133 images were randomly selected as the final test set, and the remaining 812 images were used to optimize network parameters. All image data were preprocessed in the same way as in the article. The evaluation indexes of each category in the test data set are shown in the following Table 8, and the comparison results of different base classifiers and DR-IIXRN in accuracy are shown in Figure 7. As can be seen from the data in the table and figure, the deep ensemble learning algorithm proposed in this article largely integrates the advantages of various base classifiers, the auc, accuracy, and recall rate of the proposed method are improved to 95, 92, and 92%, respectively, and the network model trained in public data sets can achieve better results after the optimization of actual data.

**Table 8:** Dataset category distribution and evaluation indicators.

**Dataset category distribution and evaluation indicators**		**0**	**1**	**2**	**3**	**4**	**Overall**
Test set	Precision	0.9	1	0.81	0.96	1	0.93
	Recall	0.95	0.69	0.97	0.89	0.97	0.92
	F1	0.93	0.81	0.89	0.92	0.99	0.92
	Specificity	0.98	1	0.91	0.99	1	0.97
	Accuracy	0.95	0.69	0.97	0.89	0.97	0.92
	AUC	0.97	0.84	0.94	0.94	0.99	0.95
	Support	20	16	36	27	34	133
Data set	Support	159	99	289	166	232	945

## 5. Conclusion

In this article, we propose a DR detection algorithm DR-IIXRN based on deep ensemble learning and attention mechanism. The experimental results show that the image preprocessing and image enhancement module can solve the problem of low classification accuracy caused by the uneven distribution of the original data. DR-IIXRN deep ensemble learning algorithm can make the base classifier play better roles in the detection of DR in actual hospitals through weight calculation. The results of the comparison with other detectors confirm the accuracy and verify the performance of the algorithm. In the future, we plan to use more actual hospital samples to test the robustness of the algorithm.

## Data Availability Statement

The datasets generated and/or analysed during the current study are available from the corresponding author on reasonable request. Correspondence and requests for data materials should be addressed to Yaping Lu (luyaping@sinopharm.com).

## Author Contributions

ZA, YF, and YL: conceptualization and writing original draft preparation. ZA and XH: methodology. ZA and FZ: writing review and editing. YL and FZ: project administration. XH and JF: data collection. YL, XH, and FZ: funding acquisition. All authors read and agreed to the published version of the manuscript.

## Funding

This work was supported in part by the National Natural Science Foundation of China to FZ (81902861) and XH (32000485) and in part by the Sinopharm Genomics Technology Co., Ltd. The funder Sinopharm Genomics Technology Co., Ltd. had the following involvement with the study: design, collection, analysis, interpretation of data, the writing of this article and the decision to submit it for publication.

## Conflict of Interest

ZA, YF, and YL are employees of Sinopharm Genomics Technology Co., Ltd. The remaining authors declare that the research was conducted in the absence of any commercial or financial relationships that could be construed as a potential conflict of interest.

## Publisher's Note

All claims expressed in this article are solely those of the authors and do not necessarily represent those of their affiliated organizations, or those of the publisher, the editors and the reviewers. Any product that may be evaluated in this article, or claim that may be made by its manufacturer, is not guaranteed or endorsed by the publisher.
